# Applicability and Eligibility of the International Study of Comparative Health Effectiveness with Medical and Invasive Approaches (ISCHEMIA) for Patients who Underwent Revascularization with Percutaneous Coronary Intervention

**DOI:** 10.3390/jcm9092889

**Published:** 2020-09-07

**Authors:** Nozomi Niimi, Mitsuaki Sawano, Nobuhiro Ikemura, Toshiyuki Nagai, Shintaro Nakano, Satoshi Shoji, Yasuyuki Shiraishi, Ikuko Ueda, Yohei Numasawa, Masahiro Suzuki, Shigetaka Noma, Keiichi Fukuda, Shun Kohsaka

**Affiliations:** 1 Department of Cardiology, Keio University School of Medicine, Tokyo 160-8582, Japan; figarofuga@gmail.com (N.N.); ikemu0129@gmail.com (N.I.); sshoji0116@gmail.com (S.S.); yasshiraishi@keio.jp (Y.S.); iueda@a7.keio.jp (I.U.); kfukuda@a2.keio.jp (K.F.); 2 Department of Cardiology, Tokyo Dental College Ichikawa General Hospital, Chiba Prefecture 272-8513, Japan; mitsuakisawano@gmail.com; 3 Department of Cardiovascular Medicine, Hokkaido University Graduate School of Medicine, Sapporo 060-8638, Japan; tnagai@huhp.hokudai.ac.jp; 4 Department of Cardiology, Saitama Medical University, International Medical Center, Saitama Prefecture 350-1298, Japan; snakano@saitama-med.ac.jp; 5 Department of Cardiology, Japanese Red Cross Ashikaga Hospital, Tochigi Prefecture 326-0843, Japan; numasawa@cpnet.med.keio.ac.jp; 6 Department of Cardiology, National Hospital Organization Saitama Hospital, Saitama Prefecture 351-0102, Japan; suzuki.masahiro.yd@mail.hosp.go.jp; 7 Department of Cardiology, Saiseikai Utsunomiya Hospital, Tochigi Prefecture 321-0974, Japan; shige_noma@chorus.ocn.ne.jp

**Keywords:** stable ischemic heart disease, ISCHEMIA, percutaneous coronary intervention, registry

## Abstract

In the International Study of Comparative Health Effectiveness with Medical and Invasive Approaches (ISCHEMIA) trial, an early invasive strategy did not decrease mortality compared to a conservative strategy for stable ischemic heart disease (SIHD) patients with moderate-to-severe ischemia, and the role of revascularization would be revised. However, the applicability and potential influence of this trial in daily practice remains unclear. Our objective was to assess the eligibility and representativeness of the ISCHEMIA trial on the patients with percutaneous coronary intervention (PCI). From a multicenter registry, we extracted a consecutive 13,223 SIHD patients with PCI (baseline cohort). We applied ISCHEMIA eligibility criteria and compared the baseline characteristics between the eligible patients and the actual study participants (randomized controlled trial (RCT) patients). In 3463 patients with follow-up information (follow-up cohort), the 2 year composite of major adverse cardiac events was evaluated between the eligible patients and RCT patients, as well as eligible and non-eligible patients in the registry. In the baseline cohort, 77.3% of SIHD patients with moderate-to-severe ischemia were eligible for the ISCHEMIA. They were comparable with RCT patients for baseline characteristics and outcomes unlike the non-eligible patients. In conclusion, the trial results seem applicable for the majority of PCI patients with moderate-to-severe ischemia except for the non-eligible patients.

## 1. Introduction

Stable ischemic heart disease (SIHD) is the leading cause of death in various countries and is responsible for a substantial proportion of healthcare costs [1]. In particular, with the widespread use of percutaneous coronary intervention (PCI), which accounts for the majority of revascularization procedures worldwide [2,3,4], annual costs associated with PCI are known to exceed US $12 billion in the US. The International Study of Comparative Health Effectiveness with Medical and Invasive Approaches (ISCHEMIA) is a recently published randomized controlled trial (RCT) that compared the conservative strategy of using optimal medical therapy (OMT) and the invasive strategy that added revascularization to OMT in patients with SIHD who had moderate-to-severe ischemia. The trial demonstrated no significant differences in the rate of all-cause death or myocardial infarction (MI) between the two strategies [5]. The clinical implication of the ISCHEMIA trial was that the SIHD patients who fulfilled the eligibility criteria and matched the profile of those in the ISCHEMIA trial can be initially treated with a conservative strategy [6]. Therefore, the indication of PCI would be reassessed post-ISCHEMIA era if the results applied.

Well conducted large-scale RCTs such as the ISCHEMIA are the gold standard to assess the efficacy of interventions and affect the recommendations in clinical practice guidelines; however, the concern is often expressed about the highly selective trial eligibility criteria. The lack of assessment of validity and eligibility is considered as a plausible explanation for the widespread underuse of the results derived from important clinical trials in routine practice, especially trials that evaluate the procedures associated with complications [7]. For example, the Systolic Blood Pressure Intervention Trial (SPRINT) [8,9] has shown that the intensive blood pressure management reduced the incidence of cardiovascular events, only 20% of patients with hypertension in the US were eligible for the trial, since patients with type 2 diabetes mellitus and a history of cerebrovascular disease were excluded [10]. Moreover, the intensive blood pressure management was concerned, which may cause adverse cardiovascular events. [11] Consequently, previous studies have shown the underuse of the results of the SPRINT trial in routine practice [12].

Due to its importance in providing recommendations for the management of patients with SIHD, the ISCHEMIA trial’s external validity has now been widely discussed [13,14,15,16]. To apply the trial results in clinical practice, the studied population must also be relevant to patients in daily practice. Furthermore, because there is a considerable number of patients who would not be eligible for RCTs in daily practice, clinicians often need data about non-eligible patients, including their prognosis [17]. However, the representativeness of the ISCHEMIA among patients with SIHD and data about non-eligible patients in clinical practice has not been sufficiently established.

The purpose of the present analysis was as follows: (1) to assess the percentage of patients who are eligible for ISCHEMIA and the reasons for ineligibility in a real-world setting, (2) to compare the baseline and long-term outcome of patients who are eligible for the ISCHEMIA in our registry with those of the actual study participants, and (3) to compare the two year prognosis according to eligibility. We used data from an inclusive, all-comer, large prospective multicenter PCI registry, the Japan Cardiovascular Database–Keio Interhospital Cardiovascular Studies (JCD–KiCS) during the period in which the ISCHEMIA was conducted. The clinical variables and outcomes in the JCD–KiCS were aligned with the data elements in the National Cardiovascular Data Registry CathPCI Registry and provided us with a unique opportunity to assess the trial results outside of the traditional network [18,19,20].

## 2. Materials and Methods

### 2.1. Data Source

The JCD–KiCS is a large, ongoing, prospective multicenter registry designed to collect the clinical data of consecutive patients undergoing PCI from 15 institutes in the Kanto area. Participating hospitals were instructed to record and register data from consecutive hospital visits for PCI using an electronic data-capturing software system equipped with a data query engine and validations to maintain data quality. The data entered were checked for completeness and internal consistency. Data quality was assured through automatic system validation and the reporting of data completeness, and through education and training for the dedicated clinical research coordinators specifically trained for the present PCI registry. These trained clinical research coordinators followed-up with all patients who consented to participate in this study. All PCI procedures were performed under the direction of the intervention team of each participating hospital according to standard care. The protocol of this study was in accordance with the principles of the Declaration of Helsinki and approved by the committee of each participating hospital, and all participants provided verbal or written consent for the baseline and follow-up data collection, separately [18].

### 2.2. Baseline Patient Cohort

**Figure 1:**
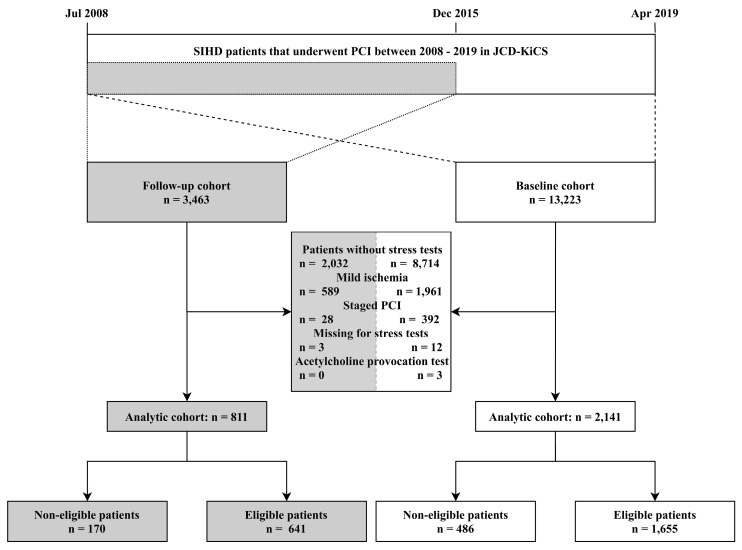
Study flow chart. Abbreviations: SIHD, stable ischemic heart disease; PCI, percutaneous coronary intervention; JCD–KiCS, The Japan Cardiovascular Database–Keio Interhospital Cardiovascular Studies.

We extracted 13,223 consecutive patients with SIHD who underwent PCI between July 2008 and April 2019 from JCD–KiCS. For the present analysis, we excluded those who did not undergo stress testing prior to PCI and those who did not meet the study inclusion criteria (Figure 1). he definition of moderate-to-severe ischemia in our registry was consistent with that of the ISCHMIA trial (details in Supplemental Table S1) [5].

The baseline characteristics of patients with and without stress tests in the baseline cohort are described in Supplemental Table S2. We then divided the patients into eligible and non-eligible groups according to each of the key ISCHEMIA exclusion criteria as follows; (i) the estimated glomerular filtration rate (eGFR) of less than 30 mL/min/1.73 m^2^ or undergoing hemodialysis, (ii) left ventricular ejection fraction (LVEF) of less than 35%, (iii) Canadian Cardiovascular Society (CCS) category IV angina patients, (iv) New York Heart Association (NYHA) classification III and IV for heart failure (HF) at admission, and (v) an unprotected left main coronary trunk (LMT) lesion. Since some of the information from the ISCHEMIA exclusion criteria was unavailable, patients meeting the following criteria were not excluded for this analysis; PCI within the previous 12 months, coronary artery bypass grafting (CABG) within 12 months, acute coronary syndrome (ACS) within the previous 2 months, hospitalization for the exacerbation of chronic HF within the previous 6 months, stroke within the previous 6 months, or a history of spontaneous intracranial hemorrhage. We handled the missing values of the main variables as follows: EF was imputed as greater than or equal to 35%, eGFR before PCI was imputed as the value of eGFR after PCI, and CCS was imputed as less than CCS IV. Other missing data were not imputed because the absence was <1% for any of the variables in our analysis.

We also measured the incidence of bleeding complications, stroke during the patient’s initial hospitalization for PCI, coronary perforation or dissection, and AKI after the PCI procedure. The post-procedural creatinine value was defined as the highest value within 30 days after the index procedure. If more than one post-procedural creatinine level was measured, the highest value was used for determining AKI. These endpoints were recorded in the database by the trained coordinators immediately after patient discharge.

### 2.3. Follow-Up Patient Cohort

For the follow-up cohort, we extracted 3463 patients from the JCD–KiCS with SIHD who agreed to participate in a long-term follow-up between July 2008 and December 2015 and to undergo screening using the aforementioned methods. The primary outcome included a composite of new onset ACS and death from a cardiovascular cause that was defined as sudden cardiac death, death from myocardial infarction, death from pulmonary embolism, and death from cerebrovascular disease within 2 years. The secondary outcome was all-cause death within 2 years. The adjudication of endpoints was carried out annually by the research coordinators independently. The median follow-up duration was 1059 days (interquartile range (IQR), 854–1313 days), and the follow-up compliance rate was 91.7%. There was no significant difference in the primary and secondary outcomes between patients with and without stress tests. (Supplemental Figure S1). The characteristics of baseline and follow-up cohort patients are described in Supplemental Table S3.

### 2.4. Statistical Analysis

#### 2.4.1. Baseline Cohort Analysis

We calculated the number and proportion of non-eligible patients and described their exclusion criteria. We compared the baseline characteristics of patients who were eligible for the ISCHEMIA from JCD–KiCS with the actual ISCHEMIA participants, [21] as well as the ISCHEMIA-eligible patients and the non-eligible patients in JCD–KiCS. Continuous variables were expressed as medians (interquartile ranges (IQRs)) and categorical variables were summarized as frequencies (%). Within the ISCHEMIA-eligible and non-eligible patients in JCD–KiCS, we compared the continuous variables using the Mann–Whitney U tests, and the categorical data using chi-squared tests or Fisher’s exact tests, as appropriate. We defined the incidence of periprocedural complications as follows: coronary artery dissection, coronary artery perforation, cardiogenic shock, heart failure, bleeding complications defined by the National Cardiovascular Data Registry CathPCI Registry [22], and acute kidney injury defined by the Acute Kidney Injury Network guideline for ≥ stage-1 renal injury, which is (i) ≥ 0.3-mg/dL absolute or ≥1.5-fold relative increase in post-PCI creatinine level, as compared with the baseline value or (ii) new initiation of dialysis [23].

#### 2.4.2. Follow-up Cohort Analysis

We compared the cumulative event rate of a composite outcome of death from cardiovascular causes and the new-onset acute coronary syndrome in ISCHEMIA-eligible patients with a composite outcome of death from cardiovascular cause and myocardial infarction in the actual ISCHEMIA participants using the Kaplan–Meier method at 6 months, 1 year, and 2 year follow-ups. Additionally, we also compared the cumulative event rate of all-cause death in these groups. Because periprocedural myocardial infarction was not included in the outcomes of our cohort, we compared the cumulative event rates during the follow-up from 6 months to 1 year and from 1 year to 2 years. Moreover, to adjust the baseline characteristics, we extracted a subgroup from the ISCHEMIA-eligible patients who were younger than 70 years old (*n* = 448), and patients who underwent OMT, defined as the prescription-based use of aspirin, P2Y12 inhibitors, and statins (*n* = 605). We compared the long-term outcomes in this subgroup of patients with the actual ISCHEMIA participants using the aforementioned methods.

Then, to assess the association between the different baseline characteristics in the baseline cohort analysis and long-term outcomes, we divided the patients who were eligible for ISCHEMIA in a long-term cohort according to those factors and compared the incidence of primary and secondary outcomes using Kaplan–Meier survival curves, the log-rank test, and performed a univariate Cox proportional hazard analysis. 

To assess the association between long-term outcomes and eligibility, we divided patients with SIHD who had moderate to severe ischemia in the long-term cohort according to their eligibility and plotted the unadjusted cumulative incidence curves using the Kaplan–Meier survival curves and compared patient groups using the log-rank test. We then performed a multivariable Cox proportional hazard analysis of patients who were not eligible for the ISCHEMIA as a reference and adjusted for age, sex, body mass index (BMI), and OMT, which were selected based on clinical significance. Additionally, we also performed a multivariable Cox model adjusted for the aforementioned covariates and mild chronic kidney disease (CKD) defined as eGFR less than 60 mL/min/1.73 m^2^ to evaluate the robustness of the model.

*p*-values < 0.05 from two-sided tests were considered statistically significant. Statistical analyses were performed using the R software (3.6.3) statistical package (Foundation for Statistical Computing, Vienna, Austria).

## 3. Results

### 3.1. Baseline Characteristics

**Figure 2:**
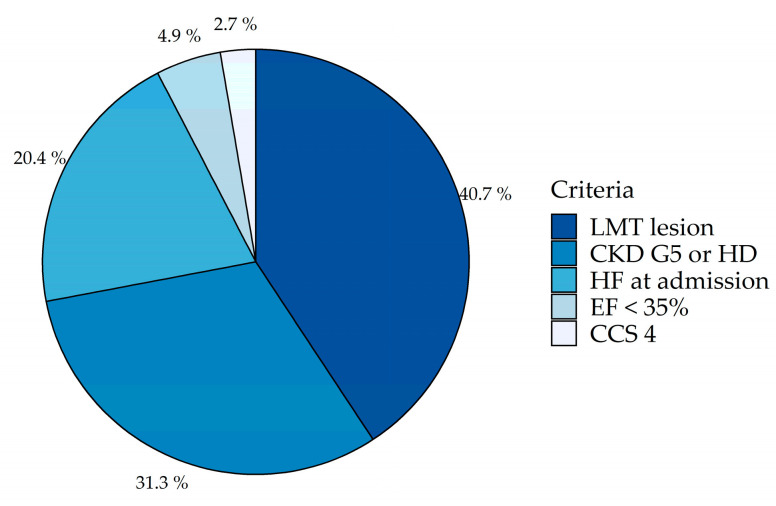
The frequency of observed exclusion criteria of the International Study of Comparative Health Effectiveness with Medical and Invasive Approaches (ISCHEMIA) trial in JCD–KiCS. Abbreviations: LMT, left main coronary trunk artery; CKD G5, chronic kidney disease grade 5; HD, hemodialysis; HF, heart failure; EF, ejection fraction; CCS, Canadian Cardiovascular Society functional classification.

Among consecutive patients with SIHD, a total of 2141 patients with proven moderate to severe ischemia (16.2% of patients with SIHD) were analyzed (Figure 1). Among these patients, a total of 1655 patients (77.3%) met the eligibility criteria for the trial. The most frequently observed exclusion criterion was the presence of an unprotected LMT lesion (198 patients; 40.7% of non-eligible patients), followed by eGFR of less than 30 mL/min/1.73 m^2^ or hemodialysis (152 patients; 31.3%), NYHA III or IV HF at admission (99 patients; 20.4%), and LVEF of less than 35% (24 patients; 4.9%) (Figure 2).

### 3.2. Trial Eligibility

The ISCHEMIA-eligible patients were largely comparable to the actual ISCHEMIA participants, albeit several discrepancies such as age, sex, previous MI, and prescription rate of beta-blockers and statins were observed. In comparison to the ISCHEMIA-eligible patients in JCD–KiCS, the non-eligible patients had high-risk features in both baseline characteristics and the coronary anatomy (Table 1).

**Figure 3:**
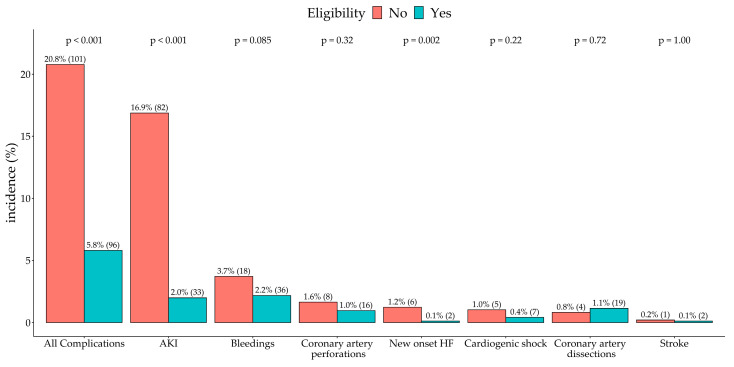
The periprocedural complications in JCD–KiCS. Abbreviations: AKI, acute kidney disease; HF, heart failure.

A total of 96 (5.8%) patients in the ISCHEMIA-eligible group and 101 (20.8%) patients in the non-eligible group experienced periprocedural complications (Figure 3).

### 3.3. Follow-up Cohort

Among 3463 SIHD patients with long-term follow-up information, a total of 811 (23.4%) patients with SIHD had moderate to severe ischemia. Of these 811 patients, 641 (79.0%) fulfilled the eligibility criteria for the ISCHEMIA trial. In the follow-up cohort, the sum of unprotected LMT lesions, HF at admission, and severe CKD were observed in more than 90% of all non-eligible patients, which was consistent with the characteristics of the baseline cohort; however, the ranking varied (Supplemental Figure S2).

The cumulative incidence rate of a composite of death from cardiovascular cause and MI in the ISCHEMIA trial was higher than that of a composite of death from cardiovascular cause and new-onset ACS in the JCD–KiCS (7.9% in the ISCHEMIA trial vs. 4.7% in JCD–KiCS at 2 year follow-up). However, after eliminating the influence of periprocedural myocardial infarction, these outcomes, as well as all-cause death, during 6 month to 1 year, and 1 year to 2 year follow-ups were comparable between the ISCHEMIA trial and JCD–KiCS (Table 2).

Additionally, in our subgroup analyses, the events rates of patients with OMT or young patients were similar to those of patients in the ISCHEMIA trial (detailed in Supplemental Table S4).

**Figure 4:**
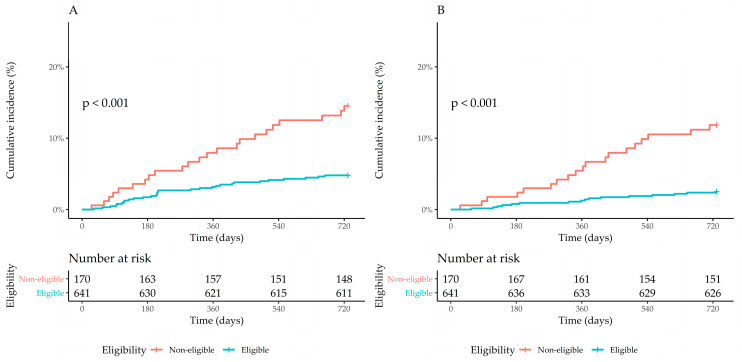
The association between long-term outcomes and eligibility: (**A**) the cumulative incidence of the composite outcome of death from cardiovascular causes and new-onset acute coronary syndrome in the eligible group and the non-eligible group; (**B**) the cumulative incidence of all-cause death in the eligible group and the non-eligible group.

The cumulative incidence of the primary outcome was 4.7% (95% confidence interval (CI): 3.0–6.3%) in patients eligible for the ISCHEMIA and 13.8% (95% CI: 8.4–18.8%) in the non-eligible patients (*p* < 0.001 for log-rank test). The cumulative incidence of the secondary outcome was also lower in eligible patients (2.5% vs. 11.4%, *p* < 0.001). The multivariable Cox regression analysis showed a significantly lower hazard ratio (HR) for both the primary (2.89; 95% CI, 1.68–4.09; *p* < 0.001) and secondary outcomes (4.50; 95% CI, 2.31–8.77; *p* < 0.001) (Figure 4 and Table 3). The results of the multivariable Cox regression model including CKD were consistent with the main findings (aHR, 2.32; 95% CI, 1.30–4.17; *p* value = 0.005 for the primary outcome; and aHR, 3.14; 95% CI, 1.54–6.38; *p* value = 0.002 for all-cause death).

**Figure 5:**
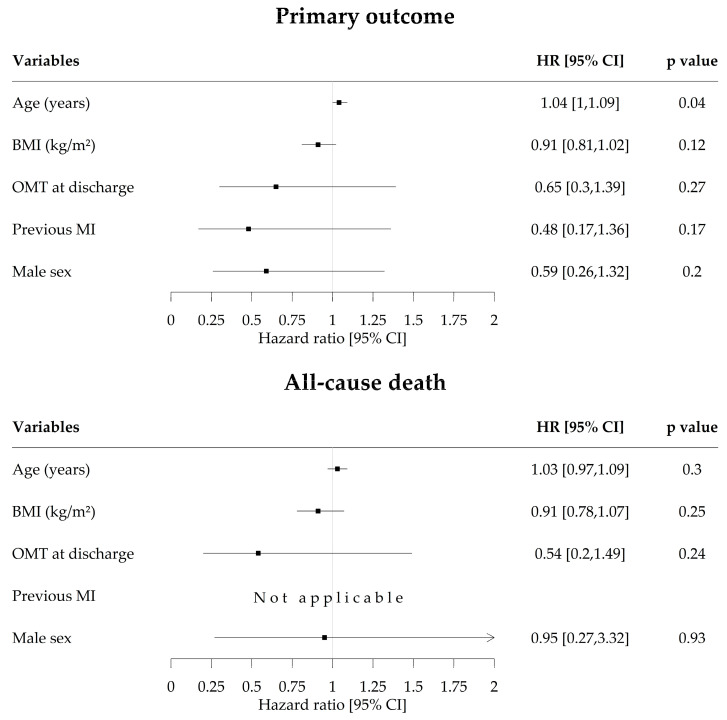
Univariate Cox regression analysis in ISCHEMIA-eligible Patients. Primary outcome was a composite of death from cardiovascular cause and the new onset acute coronary syndrome. Optimal medical therapy was defined as a prescription of aspirin, P2Y12 inhibitors, and statins after PCI. Abbreviations: BMI, body mass index; OMT, optimal medical therapy; MI, myocardial infarction; HR, hazard ratio.

Within the eligible group of patients, age was associated with a higher incidence of the primary outcome (HR for age for the primary outcome was 1.04; 95% CI, 1.00–1.09; *p* = 0.04), and previous MI was associated with a lower incidence of the secondary outcome (HR calculation not available; log-rank test *p* = 0.023) (Figure 5 and Supplemental Figure S3). In the ISCHEMIA-eligible patients, those with previous MI tended to be asymptomatic and more likely to be prescribed OMT (detailed in Supplemental Table S5).

## 4. Discussion

In this Japanese multicenter PCI cohort, we found that (1) over 75% of patients with SIHD and moderate to severe ischemia were eligible for the ISCHEMIA; (2) the ISCHEMIA participants were largely comparable to patients who met the ISCHEMIA-eligible criteria in terms of both baseline characteristics and long-term prognosis, albeit several discrepancies with the ISCHEMIA were seen in baseline characteristics such as age, sex, previous MI, and OMT; and (3) patients not eligible for the ISCHEMIA had high-risk baseline characteristics and demonstrated poor 2 year outcomes.

A previous systematic review revealed that the proportion of patients who met the eligibility criteria ranged from 10 to 70% in major cardiovascular trials [24]. Our analysis demonstrated that the majority of the patients with SIHD who underwent PCI during the study period were actually eligible for the ISCHEMIA; the high eligibility rate seen in our study may be attributed to the fewer exclusion criteria in the ISCHEMIA. The ISCHEMIA did not include an upper age limit and criteria for comorbidities, which are often used in RCTs [25]. Based on our analysis, the results of the ISCHEMIA would be highly applicable clinically and fundamental in daily clinical decision making as well as underscore that the PCI indications for most patients should be reassessed during the post-ISCHEMIA era.

Previous studies have shown that eligible patients for real-world RCTs are generally older and have more comorbidities than actual RCT participants, even without an age limit and comorbidity-related exclusion criteria [26]. Moreover, epidemiological studies have shown that Japanese patients have a relatively later onset of SIHD than Caucasians [27]. The higher age in the patients from JCD–KiCS eligible for the ISCHEMIA reflects the real-world patients with SIHD in Japan. Older patients with SIHD are frequently under-represented in clinical trials, despite the increasing prevalence of SIHD with age [1,25]. Furthermore, older patients with SIHD are known to have a more complex coronary anatomy and frequently experience complications in comparison to younger patients [28]. Therefore, the appropriate management strategy in older patients with SIHD is critical in daily practice. However, given the high hazard associated with older age in our study, caution is still needed to apply the results of the ISCHEMIA onto older patients [29]. Further studies are needed about the appropriate management strategy for the older patients with SIHD and moderate to severe ischemia. Moreover, the possible explanations for the association between prior MI and lower incidence of subsequent major adverse cardiac events were selection bias and the subsequent prescribing of OMT. Previous MI was associated with routine follow-up stress imaging tests after PCI, and that may have led to include more patients without angina that was associated with a lower incidence of major adverse cardiac events [30,31,32]. Additionally, our analysis showed that patients with prior MI (rather than stable coronary artery disease) were more likely to be receiving OMT, which was consistent with previous studies. Therefore, our results should not indicate that all SIHD patients with previous MI had better prognoses, rather to understand the possibility of the inappropriate use for asymptomatic ischemia patients and underscore the importance of the use of the OMT.

The prescription rate of OMT in the real-world setting is reported to be lower than in RCTs. For example, the prescription rate of statins was 86% in the Clinical Outcomes Utilizing Revascularization and Aggressive Drug Evaluation (COURAGE) trial, while the prescription rate of OMT was 73.3% in the Practice Innovation and Clinical Excellence (PINNACLE) registry [33]. In our analysis, as well as in the PINNACLE registry, the relatively lower prescription rate of OMT reflects the daily practice. The low OMT prescription rate may be attributed to concerns about comorbidities that may trigger a higher incidence of medication-related adverse effects. However, further studies are needed to confirm the barriers to the prescription of OMT.

When considering the extrapolation of trial results to patients that were excluded from the trial (e.g., patients with LMT lesions), it should be considered that these generally exhibit higher risk profiles and have a poor prognosis. This is in accordance with previous reports that showed that patients with unprotected LMT lesions and HF have impaired prognosis when compared to control patients [34,35,36,37]. Based on these findings, the current international clinical practice guidelines recommend revascularization for patients with unprotected LMT lesion and patients with HF, while most studies were performed prior to the era of strict OMT application [38,39].

### Study Limitations

There are several limitations to our study. First, we did not compare the actual ISCHEMIA trial participants and the patients in the JCD–KiCS eligible for the ISCHEMIA trial using statistical significance because the raw data of the ISCHEMIA trial were not available and are expressed non-parametrically. However, in daily practice, clinically significant differences are more important than statistical differences. Second, this was an observational study, so we were not able to adjust for unobserved confounders in the long-term analyses. However, our purpose was to assess the generalizability of the ISCHEMIA, rather than the causality between non-eligibility and poor prognosis. Third, we only recruited patients who underwent PCI. We did not recruit patients who underwent CABG. Additionally, more than 20% of patients in the invasive strategy arm did not undergo revascularization [5]. Therefore, we could not compare the ISCHEMIA trial participants in the invasive strategy arm (which included patients who underwent PCI and CABG) and patients who did not undergo revascularization. However, the main purpose of this analysis was not to compare the outcomes of the medical therapy, PCI, and CABG groups individually. Instead, we aimed to assess the representativeness and eligibility of the trial for patients who underwent PCI. Fourth, we did not measure the incidence of periprocedural myocardial infarction based on the Third Universal Definition of Myocardial Infarction, which is associated with the subsequent incidence of all-cause death, death from cardiovascular cause, and major adverse cardiovascular events [40,41]. In the ISCHEMIA trial, patients with type 4a MI were censored for myocardial infarction during the follow-up period from 0 to 6 months, but they were not censored in the JCD–KiCS. Therefore, we might have overestimated the incidence of myocardial infarction during the follow-up period of 6 months to 2 years in the JCD–KiCS, including high-risk type 4a MI. Fifth, we could not apply several exclusion criteria due to the unavailability of timing data. These data could have led to more patients fulfilling the exclusion criteria. Sixth, we did not accomplish all of the goals of medical therapy. However, dual antiplatelet therapy and statins are universally recommended for SIHD patients by international clinical practice guidelines because multiple large-scale, randomized controlled trials have been proven to improve the long-term prognoses of SIHD patients [38,39]. The ISCHEMIA study protocol also defined the implementation of antiplatelet therapy and statins as key elements of “optimized medical therapy”; as a result, the prescription rates of those medications were more than 95% for the study participants. At last, more than 40% of patients have not undergone stress tests before PCI. The low rate of stress tests may be explained by the fact that coronary computed tomographic angiography and/or the measurement of fractional flow reserve during invasive diagnostic angiography are common methods used to assess the indication for PCI in Japan [19].

## 5. Conclusions

In conclusion, patients with SIHD who had moderate to severe ischemia and underwent PCI in routine clinical practice were comparable to the ISCHEMIA participants for baseline and the long-term prognoses. The main results of the trial are largely applicable to real-world patients. Due to the high applicability and representativeness, the majority of PCI-eligible patients with moderate-to-severe ischemia in daily clinical practice should be reassessed in the post-ISCHEMIA era. However, caution is needed when extrapolating the trial results to non-eligible patients, since they demonstrated a higher risk of baseline characteristics and hazard in the long-term follow-up.

## Figures and Tables

**Table 1:** Baseline characteristics in JCD–KiCS, and actual ISCHEMIA participants.

Variables	Comparison between the ISCHEMIA Trial and JCD–KiCS		Comparison within the JCD–KiCS		
	Actual ISCHEMIA Participants	ISCHEMIA-Eligible Patients in the JCD–KiCS	Non-Eligible Patients	Eligible Patients	*p* Value
*n*	5179	1655	486	1655	
Background					
Age (years)	64 (58, 70)	69 (63, 75)	70 (64, 76)	69 (63, 75)	0.015
Male (%)	4011 (77.4)	1364 (83.2)	392 (81.3)	1364 (83.2)	0.37
BMI (kg/m^2^)	27.7 (25.0, 31.2)	24 (22, 27)	24 (22, 26)	24 (22, 27)	<0.001
Smoking (%)	639 (12.4)	418 (25.6)	136 (28.2)	418 (25.6)	0.27
EF (%)	60 (55, 65)	63 (55, 69)	58 (44, 67)	63 (55, 69)	<0.001
eGFR (mL/min/1.73m^2^)	82 (69, 97)	64 (54, 75)	47 (15, 64)	64 (54, 75)	<0.001
CCS (%)					<0.001
0	1039 (20.1)	609 (38.5)	178 (40.4)	609 (38.5)	
1	1384 (26.7)	247 (15.6)	64 (14.5)	247 (15.6)	
2	2524 (48.8)	591 (37.3)	131 (29.7)	591 (37.3)	
3	230 (4.4)	136 (8.6)	48 (10.9)	136 (8.6)	
4	0 (0.0)	0 (0.0)	20 (4.5)	0 (0.0)	
Hypertension (%)	3789 (73.4)	1303 (79.5)	405 (84.0)	1303 (79.5)	0.034
Diabetes mellitus (%)	2122 (41.0)	754 (46.1)	282 (58.8)	754 (46.1)	<0.001
PAD (%)	205 (4.0)	130 (7.9)	74 (15.4)	130 (7.9)	<0.001
Past medical history					
History of HF (%)	206 (4.0)	86 (5.3)	134 (27.9)	86 (5.3)	<0.001
History of stroke (%)	150 (2.9)	147 (9.0)	69 (14.3)	147 (9.0)	0.001
History of MI (%)	990 (19.2)	507 (31.0)	176 (36.5)	507 (31.0)	0.025
History of PCI (%)	1050 (20.3)	804 (49.0)	261 (54.1)	804 (49.0)	0.053
History of CABG (%)	200 (3.9)	98 (6.0)	73 (15.1)	98 (6.0)	<0.001
Angiographic characteristics					
Proximal LAD (%)	1749 (46.8)	569 (34.7)	221 (45.8)	569 (34.7)	<0.001
LCX lesion (%)	2354 (67.4)	867 (52.9)	323 (66.7)	867 (52.9)	<0.001
RCA lesion (%)	2311 (68.8)	876 (53.9)	305 (63.9)	876 (53.9)	<0.001
Multivessel disease (%)	2679 (79.0)	1029 (62.2)	408 (84.0)	1029 (62.2)	<0.001
Medication at discharge					
Aspirin (%)	4871 (94.1)	1613 (98.7)	471 (97.7)	1613 (98.7)	0.17
RAASi (%)	3413 (66.0)	960 (58.8)	319 (66.2)	960 (58.8)	0.004
Beta blockers (%)	4161 (80.4)	1129 (69.1)	361 (74.9)	1129 (69.1)	0.017
Statins (%)	4904 (94.8)	1419 (86.8)	389 (80.7)	1419 (86.8)	0.001

Data presented as median (interquartile range (IQR)) or n (%). Abbreviations: BMI, body mass index; EF, ejection fraction; eGFR, estimated glomerular filtration rate; CCS, Canadian Cardiovascular Society functional classification; PAD, peripheral artery disease; HF, heart failure; MI, myocardial infarction; PCI, percutaneous coronary intervention; CABG, coronary artery bypass grafting; LAD, left anterior descending; LCX, left circumflex artery; RCA, right coronary artery; RAASi, renin–angiotensin–aldosterone system inhibitors; ISCHEMIA, International Study of Comparative Health Effectiveness with Medical and Invasive Approaches trial; JCD–KiCS, The Japan Cardiovascular Database–Keio Interhospital Cardiovascular Studies.

**Table 2:** Long-term outcomes of the actual ISCHEMIA participants and the ISCHEMIA-eligible patients in JCD–KiCS.

Outcome	Time	Patients in Invasive Strategy in the ISCHEMIA Trial (%)	ISCHEMIA-Eligible Patients in JCD–KiCS (95% CI) (%)
Primary outcome	0 to 6 month	4.8	1.7 (0.7–2.7)
	6 month to 1 year	1.4	1.4 (0.5–2.4)
	1 year to 2 year	1.7	1.6 (0.6–2.6)
All-cause death	0 to 6 month	0.8	0.8 (0.1–1.5)
	6 month to 1 year	0.9	0.5 (0.0–1.0)
	1 year to 2 year	1.1	1.3 (0.4–2.1)

Events rate was obtained by the Kaplan–Meier method. Primary outcome was a composite of death from cardiovascular cause and myocardial infarction in the ISCHEMIA trial and a composite of death from cardiovascular cause and acute coronary syndrome in JCD–KiCS. Abbreviations: ISCHEMIA, International Study of Comparative Health Effectiveness with Medical and Invasive Approaches trial; JCD–KiCS, The Japan Cardiovascular Database–Keio Interhospital Cardiovascular Studies; CI, confidence interval.

**Table 3:** Clinical outcomes and hazard ratios according to eligibility.

Endpoint	Eligible Patients (*n* = 641)	Non-Eligible Patients (*n* = 170)	aHR (95% CI)	*p* Value
	Events Rate (%)	Events Rate (%)		
Primary outcome	4.7 (3–6.3)	13.5 (8.2–18.5)	2.89 (1.68–4.98)	<0.001
All-cause death	2.5 (1.3–3.7)	11.2 (6.3–15.8)	4.5 (2.31–8.77)	<0.001

Events rate was obtained by the Kaplan–Meier method and hazard ratio was calculated by Cox hazard model adjusted by age, sex, body mass index, and optimal medical therapy defined as dual antiplatelet therapy and statins at discharge. The primary outcome was a composite of death from cardiovascular cause and new onset of acute coronary syndromes. Abbreviations: CI, confidence interval; aHR, adjusted hazard ratio.

## Supplementary Materials

The following are available online at https://www.mdpi.com/2077-0383/9/9/2889/s1, Figure S1: The association between long-term outcomes stratified by performed stress tests before PCI in JCD–KiCS, Figure S2: The frequency of the observed exclusion criteria of the ISCHEMIA trial in follow-up cohort, Figure S3: Kaplan–Meier curve in subgroups of the ISCHEMIA-eligible patients in JCD–KiCS, Table S1: Baseline characteristics stratified by stress test performed before PCI in JCD–KiCS, Table S2: Baseline characteristics of patients in baseline cohort and follow-up cohort, Table S3: Long-term analysis in a subgroup of JCD–KiCS, Table S4: Long-term analysis in subgroup JCD–KiCS, Table S5: Baseline characteristics of patients in with and without prior MI.
