# Supplementary material for: Applicability and Eligibility of the International Study of Comparative Health Effectiveness with Medical and Invasive Approaches (ISCHEMIA) for Patients who Underwent Revascularization with Percutaneous Coronary Intervention
Source: J Clin Med. 2020 Sep 7;9(9):2889. doi: 10.3390/jcm9092889 (PMC7564619; doi:10.3390/jcm9092889)
Supplement: Supplementary file 1 [file jcm-09-02889-s001.zip › suppelemental/supplemental_figures/suppelemtal_figures.docx]

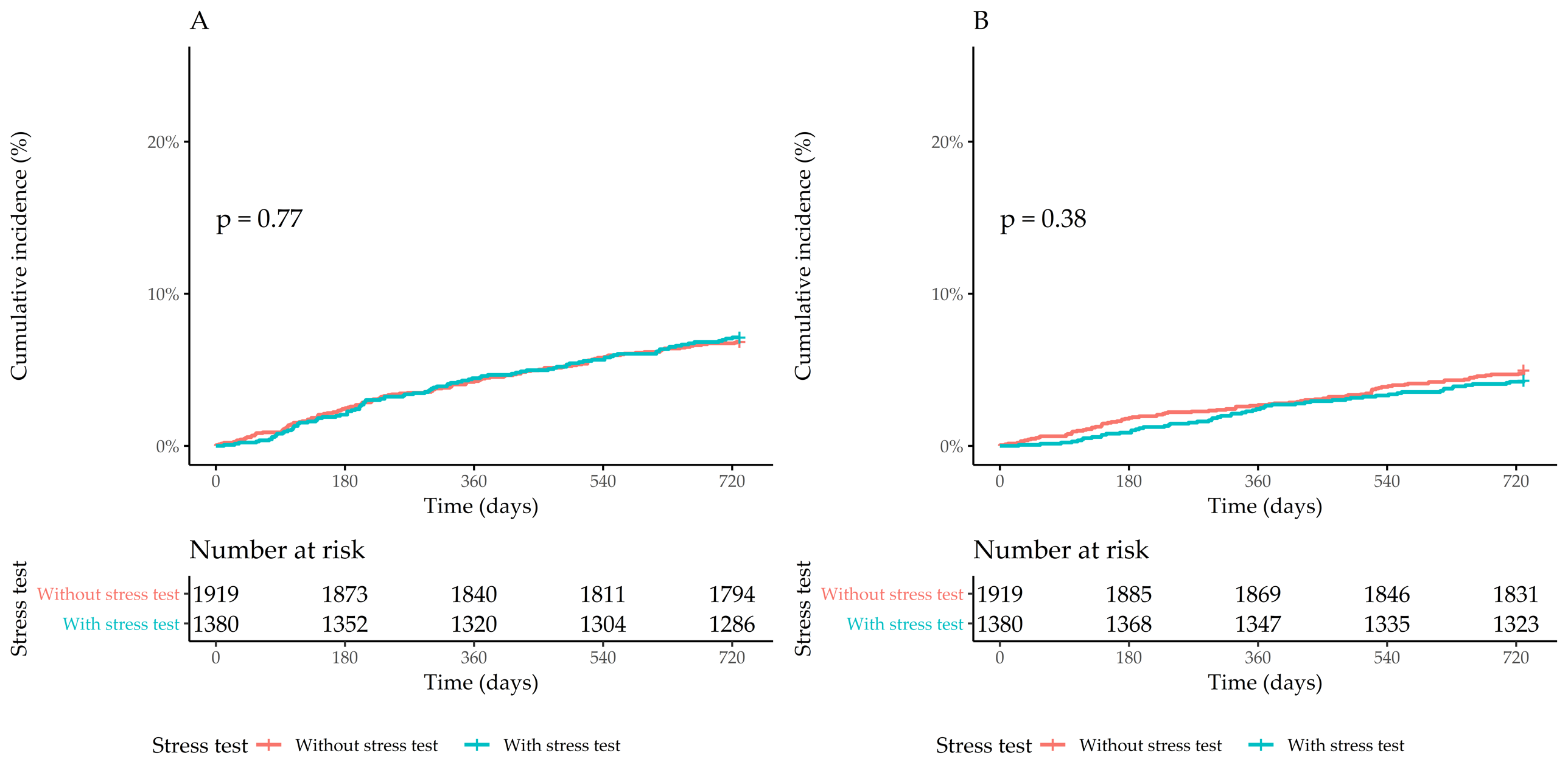


**Supplemental Figure 1.** The Association Between Long-term Outcomes stratified by Performed Stress Tests Before PCI in JCD-KiCS. (A) The cumulative incidence of the composite outcome of death from cardiovascular causes and new-onset acute coronary syndrome in the patients with and without non-stress tests before PCI; (B) The cumulative incidence of all-cause death in the patients with and without non-stress tests before PCI.


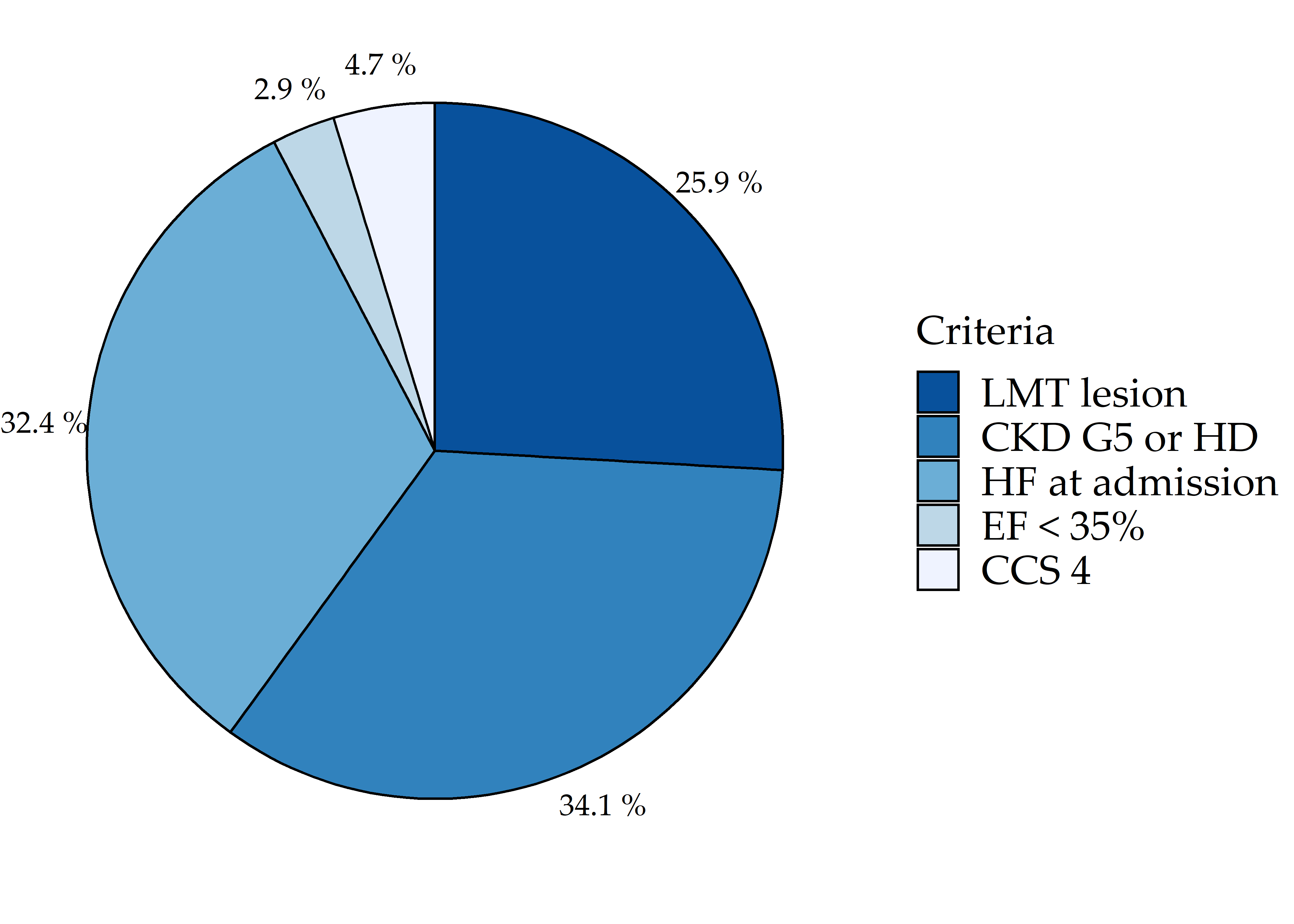


**Supplemental Figure 2.** The Frequency of observed Exclusion Criteria of the ISCHEMIA trial in follow-up cohort. Abbreviations: LMT, left main coronary trunk artery; CKD G5, chronic kidney disease grade 5; HD, hemodialysis; HF, heart failure; EF, ejection fraction; CCS, Canadian Cardiovascular Society functional classification.





**Supplemental Figure 3.** Kaplan-Meier Curve in Subgroups of the ISCHEMIA-eligible Patients in JCD-KiCS. Primary outcome was a composite of death from cardiovascular cause and new onset acute coronary syndrome. Optimal medical therapy was defined as prescription of aspirin, P2Y12 inhibitors, and statins after PCI. Abbreviations: BMI, body mass index; MI, myocardial infarction; OMT, optimal medical therapy.
