# Supplementary material for: Applicability and Eligibility of the International Study of Comparative Health Effectiveness with Medical and Invasive Approaches (ISCHEMIA) for Patients who Underwent Revascularization with Percutaneous Coronary Intervention
Source: J Clin Med. 2020 Sep 7;9(9):2889. doi: 10.3390/jcm9092889 (PMC7564619; doi:10.3390/jcm9092889)
Supplement: Supplementary file 1 [file jcm-09-02889-s001.zip › suppelemental/supplemental_tables/suppelementa_table.docx]

| Supplemental Table 1. Baseline characteristics stratified by stress test performed before PCI in JCD-KiCS. | | | |
| --- | --- | --- | --- |
| Variables | Without stress test | With stress test | p value |
| n | 6022 | 3792 |  |
| Background |  |  |  |
| Age (years) | 70 [63, 77] | 70 [63, 76] | <0.001 |
| Male (%) | 4691 (79.1) | 3052 (81.1) | 0.015 |
| BMI (kg/m²) | 24 [22, 26] | 24 [22, 27] | <0.001 |
| Current smoker (%) | 1508 (25.5) | 933 (24.8) | 0.480 |
| Ejection fraction (%) | 62 [50, 68] | 63 [54, 70] | <0.001 |
| eGFR (ml/min/1.73m²) | 61 [47, 73] | 62 [50, 73] | <0.001 |
| Hypertension (%) | 4586 (77.4) | 2985 (79.4) | 0.026 |
| Diabetes Mellitus (%) | 2781 (47.0) | 1793 (47.8) | 0.476 |
| PAD (%) | 805 (13.6) | 396 (10.5) | <0.001 |
| CCS (%) |  |  | <0.001 |
| 0 | 1990 (36.0) | 1449 (40.4) |  |
| 1 | 1065 (19.3) | 609 (17.0) |  |
| 2 | 1827 (33.0) | 1176 (32.8) |  |
| 3 | 576 (10.4) | 312 (8.7) |  |
| 4 | 74 (1.3) | 39 (1.1) |  |
| Past history of HF (%) | 847 (14.3) | 339 (9.0) | <0.001 |
| Past history of stroke (%) | 579 (9.8) | 352 (9.4) | 0.523 |
| Past history of MI (%) | 1571 (26.5) | 1130 (30.0) | <0.001 |
| Past history of PCI (%) | 2514 (42.4) | 1863 (49.5) | <0.001 |
| Past history of CABG (%) | 422 (7.1) | 268 (7.1) | 1.000 |
| HF at admission (%) | 678 (11.3) | 189 (5.0) | <0.001 |
| Non-invasive tests |  |  |  |
| MPI (%) | 0 (0.0) | 2773 (73.1) | <0.001 |
| Treadmill test (%) | 0 (0.0) | 1221 (32.2) | <0.001 |
| CMR (%) | 0 (0.0) | 57 (1.5) | <0.001 |
| CCTA (%) | 2533 (42.1) | 1362 (35.9) | <0.001 |
| FFR (%) | 275 (4.6) | 208 (5.5) | 0.045 |
| Angiographic characteristics |  |  |  |
| Unprotected LMT lesion (%) | 666 (11.2) | 369 (9.9) | 0.038 |
| proximal LAD lesion (%) | 2269 (38.1) | 1339 (35.7) | 0.016 |
| LCX lesion (%) | 3006 (50.6) | 1981 (52.8) | 0.038 |
| RCA lesion (%) | 3242 (55.0) | 2008 (54.0) | 0.363 |
| Multivessel disease (%) | 3855 (64.0) | 2439 (64.3) | 0.776 |
| Medication at discharge |  |  |  |
| Aspirin (%) | 5832 (98.9) | 3707 (98.8) | 0.620 |
| RAASi (%) | 3462 (58.7) | 2215 (59.1) | 0.777 |
| beta blockers (%) | 3703 (62.8) | 2546 (67.9) | <0.001 |
| Statins (%) | 4901 (83.2) | 3185 (84.9) | 0.025 |
| High volume center (%) | 5028 (83.5) | 2958 (78.0) | <0.001 |
| Data presented as median [interquartile range (IQR)] or n (%). Abbreviations: BMI, body mass index; EF, ejection fraction; eGFR, estimated glomerular filtration rate; PAD, peripheral artery disease; CCS, Canadian Cardiovascular Society functional classification; HF, heart failure; MI, myocardial infarction; PCI, percutaneous coronary intervention; CABG, coronary artery bypass grafting; MPI, myocardial perfusion imaging; CMR, cardiovascular magnetic resonance imaging: CCTA, coronary computed tomography angiography; FFR, fractional flow reserve; LMT, left main coronary trunk; LAD, left anterior descending; LCX, left circumflex artery; RCA, right coronary artery; RAASi, renin-angiotensin-aldosterone system inhibitors. | | | |

| Supplemental Table 2. Baseline Characteristics of Patients in Baseline Cohort and Follow-up Cohort | | | |
| --- | --- | --- | --- |
| Variables | Follow-up cohort | Baseline cohort | p value |
| n | 811 | 2141 |  |
| Background |  |  |  |
| Age (years) | 69 [62, 75] | 69 [63, 75] | 0.84 |
| Male (%) | 659 (81.4) | 1756 (82.8) | 0.39 |
| BMI (kg/m²) | 24 [22, 26] | 24 [22, 26] | 0.40 |
| Smoking (%) | 225 (27.8) | 554 (26.2) | 0.39 |
| EF (%) | 63 [53, 69] | 62 [53, 69] | 0.60 |
| eGFR (ml/min/1.73m²) | 63 [50, 74] | 62 [49, 73] | 0.39 |
| Hypertension (%) | 656 (81.0) | 1708 (80.6) | 0.84 |
| Diabetes Mellitus (%) | 374 (46.4) | 1036 (49.0) | 0.22 |
| PAD (%) | 80 (9.9) | 204 (9.6) | 0.89 |
| Past medical history |  |  |  |
| Past history of HF (%) | 82 (10.1) | 220 (10.4) | 0.90 |
| Past history of stroke (%) | 78 (9.6) | 216 (10.2) | 0.70 |
| Past history of MI (%) | 219 (27.0) | 683 (32.2) | 0.008 |
| Past history of PCI (%) | 278 (34.3) | 1065 (50.2) | < 0.001 |
| Past history of CABG (%) | 64 (7.9) | 171 (8.1) | 0.95 |
| Angiographic characteristics |  |  |  |
| proximal LAD lesion (%) | 318 (39.4) | 790 (37.2) | 0.31 |
| LCX lesion (%) | 418 (51.7) | 1190 (56.1) | 0.038 |
| RCA lesion (%) | 409 (50.9) | 1181 (56.2) | 0.012 |
| Multivessel disease (%) | 510 (62.9) | 1437 (67.1) | 0.034 |
| Medication at discharge |  |  |  |
| Aspirin (%) | 788 (97.6) | 2084 (98.5) | 0.16 |
| RAASi (%) | 486 (60.2) | 1279 (60.4) | 0.95 |
| Beta blockers (%) | 539 (66.7) | 1490 (70.4) | 0.057 |
| Statin at discharge (%) | 676 (83.8) | 1808 (85.4) | 0.29 |
| Data given as median [interquartile range (IQR)] or n (%).  Abbreviations: BMI, body mass index; EF, ejection fraction; eGFR, estimated glomerular filtration rate; PAD, peripheral artery disease; HF, heart failure; MI, myocardial infarction; PCI, percutaneous coronary intervention; CABG, coronary artery bypass grafting; LAD, left anterior descending; LCX, left circumflex artery; RCA, right coronary artery; RAASi, renin-angiotensin-aldosterone system inhibitors; ISCHEMIA, International Study of Comparative Health Effectiveness with Medical and Invasive Approaches trial | | | |

| Supplemental Table 3. Long-term analysis in a subgroup of JCD-KiCS. | | | | | | |
| --- | --- | --- | --- | --- | --- | --- |
| outcome | Time | Patients in Invasive strategy in the ISCHEMIA trial (%) | ISCHEMIA eligible patients in JCD-KiCS | | | |
|  |  |  | Age ≤ 70 years  n = 448  (95% CI) (%) | Age > 70 years  n = 363  (95% CI) (%) | OMT  n = 605  (95% CI) (%) | non-OMT  n = 206  (95% CI) (%) |
| Primary outcome | 0 to 6-month | 4.8 | 1.1 (0.0 - 2.2) | 2.5 (0.6 - 4.3) | 1.9 (0.6 - 3.1) | 1.3 (0.0 - 3.0) |
|  | 6-month to 1-year | 1.4 | 0.3 (0.0 - 0.8) | 2.9 (0.9 - 4.9) | 0.6 (0.0 - 1.3) | 3.8 (0.8 - 6.8) |
|  | 1-year to 2-year | 1.7 | 1.1 (0.0 - 2.2) | 2.2 (0.5 - 4.0) | 1.7 (0.5 - 2.9) | 1.3 (0.0 - 3.2) |
| All-cause death | 0 to 6-month | 0.8 | 0.8 (0.0 - 1.8) | 0.7 (0.0 - 1.7) | 0.8 (0.0 - 1.6) | 0.6 (0.0 - 1.9) |
|  | 6-month to 1-year | 0.9 | 0.3 (0.0 - 0.8) | 0.7 (0.0 - 1.7) | 0.0 (0.0 - 0.0) | 1.9 (0.0 - 4.0) |
|  | 1-year to 2-year | 1.1 | 0.6 (0.0 - 1.3) | 2.2 (0.4 - 3.8) | 1.3 (0.3 - 2.2) | 1.3 (0.0 - 3.1) |
| Events rate was obtained by the Kaplan-Meier method. Primary outcome was a composite of death from cardiovascular cause and myocardial infarction in ISCHEMIA trial and a composite of death from cardiovascular cause and acute coronary syndrome in JCD-KiCS. optimal medical therapy was defined as prescription of aspirin, P2Y12 inhibitors, and statins after PCI. Abbreviations: JCD-KiCS, The Japan Cardiovascular Database-Keio Interhospital Cardiovascular Studies; ISCHEMIA, International Study of Comparative Health Effectiveness with Medical and Invasive Approaches trial; OMT, optimal medical therapy. | | | | | | |

| Supplemental table 5. Baseline Characteristics of Patients in With and Without prior MI | | | |
| --- | --- | --- | --- |
| Variables | Patients Without previous MI | Patients With previous MI | p value |
| n | 486 | 155 |  |
| Background |  |  |  |
| Age (years) | 69 [62, 75] | 69 [62, 76] | 0.61 |
| Male (%) | 394 (81.1) | 132 (85.2) | 0.30 |
| BMI (kg/m²) | 24 [22, 26] | 24 [22, 26] | 0.68 |
| Smoking (%) | 127 (26.1) | 44 (28.6) | 0.62 |
| EF (%) | 65 [59, 72] | 58 [50, 67] | < 0.001 |
| eGFR (ml/min/1.73m²) | 64 [54, 76] | 64 [55, 77] | 0.98 |
| Hypertension (%) | 390 (80.2) | 117 (75.5) | 0.25 |
| Diabetes mellitus (%) | 212 (43.9) | 72 (46.8) | 0.60 |
| PAD (%) | 40 (8.2) | 12 (7.7) | 0.98 |
| CCS (%) |  |  | 0.051 |
| 0 | 27 (8.2) | 11 (14.7) |  |
| 1 | 77 (23.3) | 23 (30.7) |  |
| 2 | 182 (55.2) | 37 (49.3) |  |
| 3 | 44 (13.3) | 4 (5.3) |  |
| Past history of HF (%) | 13 (2.7) | 15 (9.7) | < 0.001 |
| Past history of stroke (%) | 42 (8.6) | 15 (9.7) | 0.82 |
| Past history of PCI (%) | 94 (19.3) | 112 (72.3) | < 0.001 |
| Past history of CABG (%) | 18 (3.7) | 18 (11.6) | < 0.001 |
| Angiographic characteristics |  |  |  |
| Proximal LAD lesion (%) | 180 (37.2) | 60 (39.0) | 0.76 |
| LCX lesion (%) | 224 (46.3) | 88 (57.1) | 0.024 |
| RCA lesion (%) | 223 (46.3) | 86 (56.6) | 0.034 |
| Multivessel disease (%) | 268 (55.1) | 105 (67.7) | 0.007 |
| Medication at discharge |  |  |  |
| Aspirin (%) | 472 (97.5) | 150 (97.4) | 1.00 |
| RAASi (%) | 251 (51.9) | 114 (74.0) | < 0.001 |
| Beta blockers (%) | 291 (60.0) | 122 (79.2) | < 0.001 |
| Statins (%) | 397 (82.0) | 138 (89.6) | 0.035 |
| High volume center (%) | 289 (59.5) | 95 (61.3) | 0.76 |
| Data presented as median [interquartile range (IQR)] or n (%). Abbreviations: MI, myocardial infarction; BMI, body mass index; EF, ejection fraction; eGFR, estimated glomerular filtration rate; PAD, peripheral artery disease; CCS, Canadian Cardiovascular Society functional classification; HF, heart failure; PCI, percutaneous coronary intervention; CABG, coronary artery bypass grafting; LAD, left anterior descending; LCX, left circumflex artery; RCA, right coronary artery; RAASi, renin-angiotensin-aldosterone system inhibitors. | | | |
