# Supplementary figures and images for: Applicability and Eligibility of the International Study of Comparative Health Effectiveness with Medical and Invasive Approaches (ISCHEMIA) for Patients who Underwent Revascularization with Percutaneous Coronary Intervention
Source: J Clin Med. 2020 Sep 7;9(9):2889. doi: 10.3390/jcm9092889 (PMC7564619; doi:10.3390/jcm9092889)

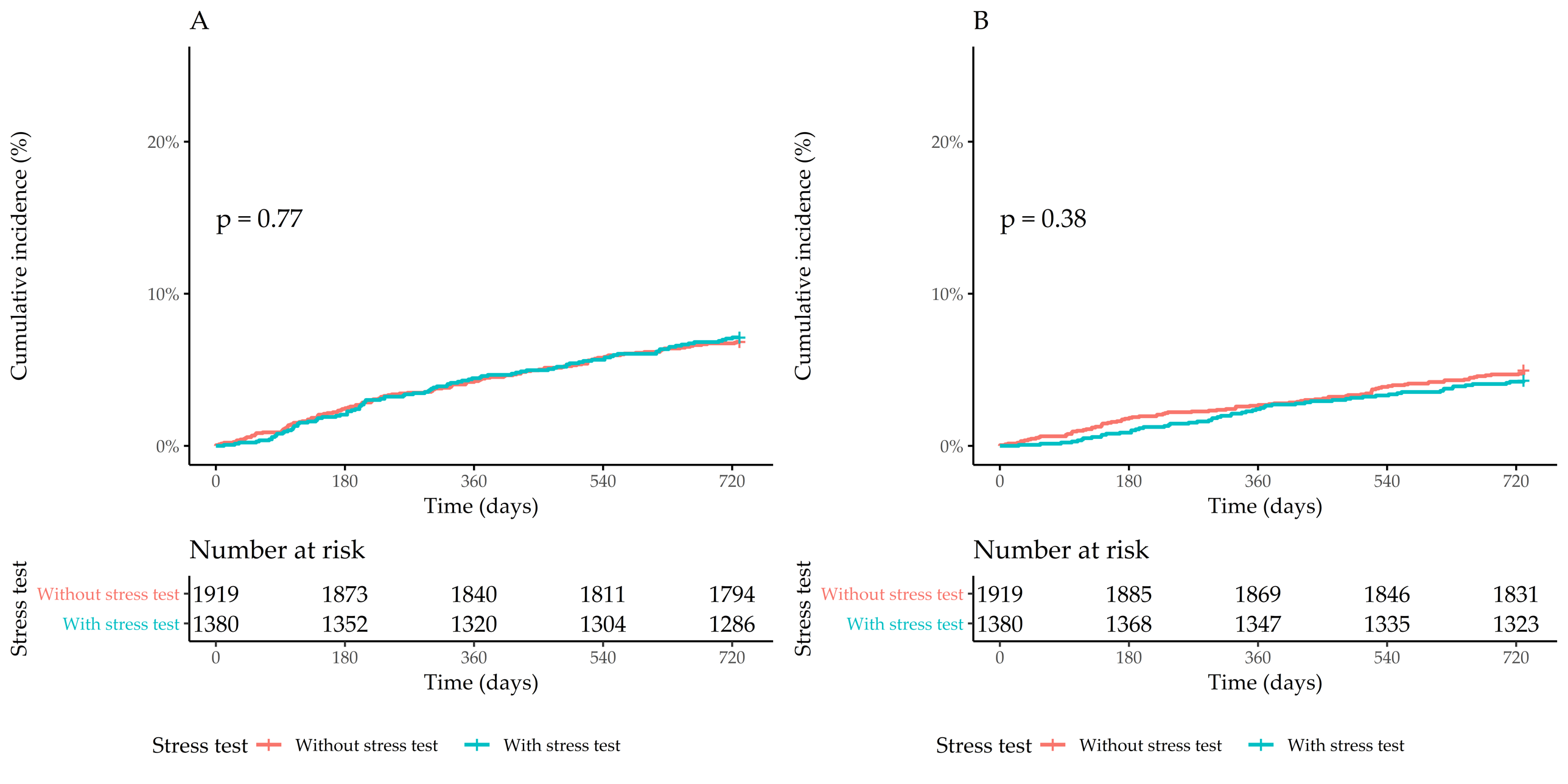

Supplement: Supplementary file 1 [file jcm-09-02889-s001.zip › suppelemental/supplemental_figures/supp_fig1_km_curve_pretest.tiff]

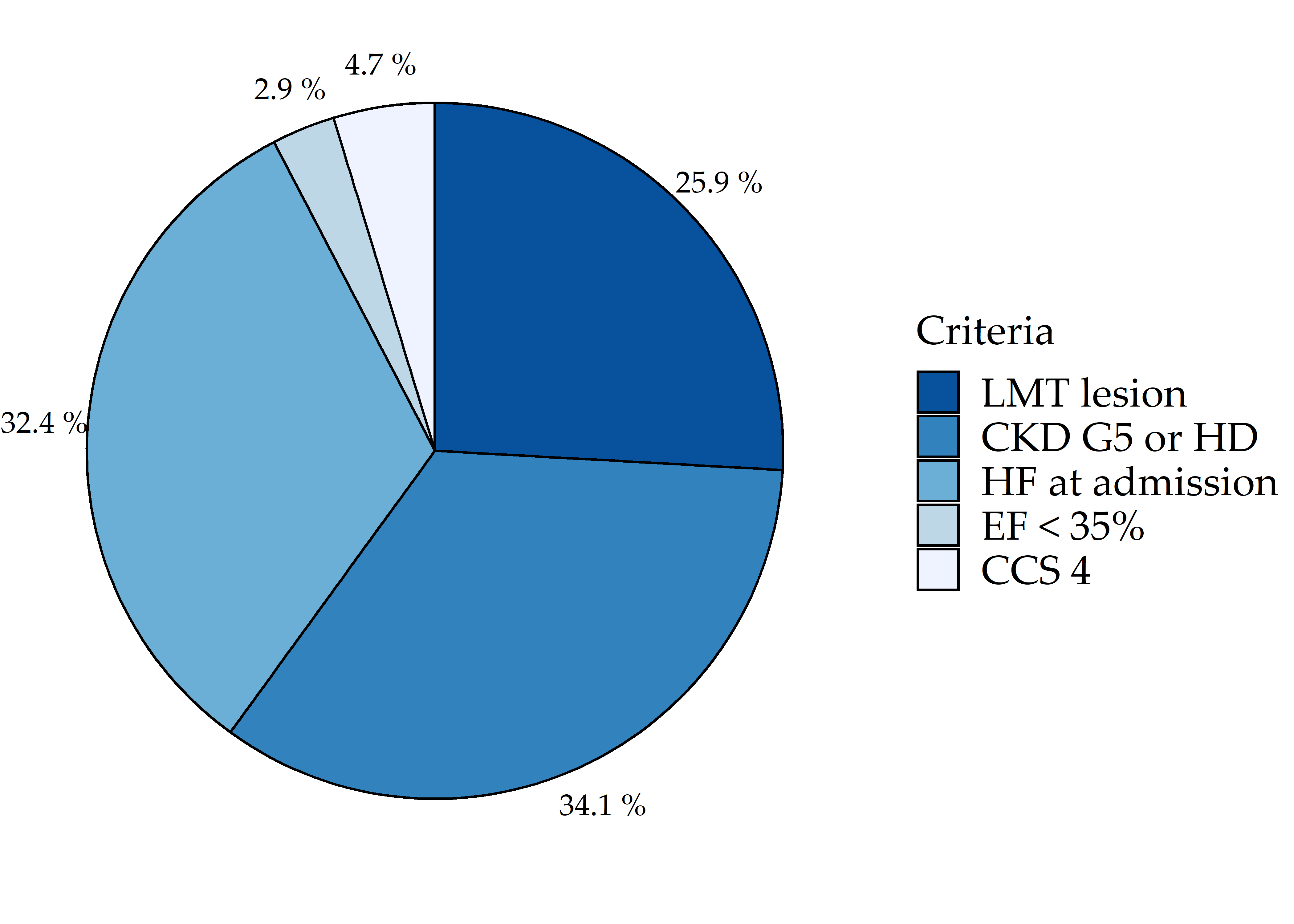

Supplement: Supplementary file 1 [file jcm-09-02889-s001.zip › suppelemental/supplemental_figures/supp_fig2_exclude_pie_long.tiff]
